# External Assessment of the Anti-N-Methyl-D-Aspartate Receptor Encephalitis One-Year Functional Status Score in Chinese Pediatric Patients

**DOI:** 10.3389/fimmu.2022.889394

**Published:** 2022-06-23

**Authors:** Hanyu Luo, Yuhang Li, Yaxin Zheng, Lvli Zhou, Jiaxin Yang, Zhixu Fang, Yan Jiang, Juan Wang, Zhengxiong Yao, Min Chen, Li Jiang

**Affiliations:** Department of Neurology, Children’s Hospital of Chongqing Medical University, National Clinical Research Center for Child Health and Disorders, Ministry of Education Key Laboratory of Child Development and Disorders, Chongqing Key Laboratory of Pediatrics, Chongqing, China

**Keywords:** anti-NMDAR encephalitis, functional status, outcome, prediction, children

## Abstract

**Objective:**

to assess the performance of the Anti-N-Methyl-D-Aspartate Receptor encephalitis (NMDAR) One-Year Functional Status (NEOS) score in predicting one-year functional outcome in Chinese children with anti-NMDAR encephalitis.

**Methods:**

children with anti-NMDAR encephalitis at the Children’s Hospital of Chongqing Medical University were retrospectively enrolled from January 2014 to December 2020. Patients were categorized into two groups based on the modified Rankin Scale (mRS) at one-year follow-up. Discrimination of the NEOS score was assessed by the area under curve (AUC) of the receiver operating characteristic curve. Calibration of the NEOS score was assessed by comparing predicted probabilities with observed probabilities using a calibration curve and the Hosmer-Lemeshow test. The clinical practicability of the NEOS score was evaluated by performing a decision curve analysis.

**Results:**

one hundred seventy-five children (101 females and 74 males) with anti-NMDAR encephalitis and a median age of 7.7 years were enrolled. Of those, 149 (85.1%) had a good outcome at 1 year (mRS ≤ 2), and the remaining 26 (14.9%) had a poor outcome (mRS > 2). Patients with a higher NEOS score had a significantly higher mRS at one-year follow-up [Spearman r = 0.3878, 95% confidence interval (CI): 0.2500-0.5103, P < 0.001]. The AUC of the NEOS score was 0.870 (95% CI: 0.801-0.938, P < 0.001). The observed probability and predicted probability showed moderate consistency in the calibration curve and the Hosmer-Lemeshow test (P = 0.912). The decision curve analysis showed that using the NEOS score to predict one-year outcomes could provide additional net benefit during clinical practice.

**Conclusions:**

the NEOS score is a potentially reliable model to predict the one-year functional outcome in Chinese children with anti-NMDAR encephalitis.

## Introduction

Anti-N-methyl-D-aspartate receptor (NMDAR) encephalitis is the most common autoimmune encephalitis caused by an antibody-mediated autoimmune response against the GluN1 subunit of the receptor. Clinical manifestations include psychiatric disorders, cognitive dysfunction, speech dysfunction, seizures, movement disorder, autonomic dysfunction and central hypoventilation (1). Most patients respond well to first-line immunotherapy including intravenous immunoglobulin, methylprednisone, or plasma exchange (2). Early initiation of second-line or enhanced immunotherapy could improve the outcome. Children with anti-NMDAR encephalitis usually have a favorable prognosis, but some patients have neurological deficits with a mortality of 4% (3). Thus, it is crucial for clinicians to identify children with severe anti-NMDAR encephalitis who may have an increased risk for poor outcomes.

Several clinical factors or biomarkers have been reported to be potential predictors of outcome in patients with anti-NMDAR encephalitis (4). However, their clinical applicability still required further validation. In 2019, Balu et al. developed a novel model (5), termed the anti-NMDAR Encephalitis One-Year Functional Status (NEOS) score, to predict one-year functional outcome of patients using five common clinical values including ICU admission, the absence of treatment for more than 4 weeks, improvement delay of more than 4 weeks after immunotherapy, abnormal MRI and a cerebrospinal fluid (CSF) white blood cell (WBC) count of > 20 cells/μL (**Table 1**). This model showed great accuracy in the original cohorts, and its performance was subsequently validated in a prospective cohort of Chinese patients with anti-NMDAR encephalitis (6). However, the clinical characteristics of patients with anti-NMDAR encephalitis could be age-related, in that children may have different risk factors for poor functional outcome compared to adult patients (7–9). Thus, it is still necessary to evaluate the applicability of the NEOS score in children.

**Table 1:** The anti-N-Methyl-D-Aspartate Receptor (NMDAR) Encephalitis One-Year Functional Status (NEOS) score.

Variables	Score
ICU admission required	1
No clinical improvement after 4 weeks of treatment	1
No treatment within 4 weeks of symptom onset	1
Abnormal MRI	1
CSF WBC count > 20 cells/μL	1

In this study, we evaluated the performance of the NEOS score in Chinese children with anti-NMDAR encephalitis to help early identification of pediatric patients with a high probability of poor neurological outcome.

## Materials and Methods

### Patients and Study Design

Children with anti-NMDAR encephalitis at the Children’s Hospital of Chongqing Medical University were retrospectively reviewed from January 2014 to December 2020. The inclusion criteria were as follows (10): (a) acute onset of one or more of the following six major groups of manifestations: abnormal (psychiatric) behaviors or cognitive dysfunctions, speech dysfunctions, seizures, movement disorders, decreased consciousness levels, autonomic dysfunction, or central hypoventilation; (b) tested positive for NMDAR IgG in CSF using cell-based assay; (c) reasonable exclusion of other disorders; (d) age between 0 and 18 years. Patients were excluded from our study if: (a) they did not undergo immunotherapy; (b) their records lacked data on the five values of the NEOS score; (c) they were lost to follow-up. Clinical data including the five items of the NEOS score and other characteristics were extracted from medical records. No clinical improvement 4 weeks after immunotherapy was defined as no improvement on the modified Rankin Scale (mRS) or mRS ≥ 4 four weeks after the initiation of immunotherapy. The functional status at one year was assessed by the mRS and the Pediatric Cerebral Performance Category (PCPC) scale (11, 12). The mRS and PCPC scale were independently determined by two pediatric neurologists based on medical records from routine clinical visits or *via* telephone. Another senior pediatric neurologist was consulted for a final decision when disagreement exited. Patients with a mRS ≤ 2 were considered to have a good functional outcome.

This study was approved by the ethics committees of the Children’s Hospital of Chongqing Medical University. Informed consent was obtained from patient’s parents.

### Statistical Analysis

Continuous variables were presented as mean and standard deviation (SD) or median and interquartile range (IQR) when the distribution was not normal. Differences were tested using Student’s t-test or the Mann-Whitney U test. Categorical variables were expressed as percentages and compared by the χ^2^ test or Fisher’s exact test. A P value ≤ 0.05 in a two-tailed test was considered statistically significant.

Odds ratio and Spearman correlation were used to measure the relationship between the one-year outcome and the NEOS score. We assessed the performance of the NEOS score in predicting the one-year outcomes of children with anti-NMDAR encephalitis by determining the discrimination and calibration. Discriminative performance is the ability to distinguish between patients with a good and a poor outcome, and this was quantified by the area under the curve (AUC) of the receiver operating characteristic curve (ROC). Calibration performance is the predicted accuracy between predicted probability and observed events of the NEOS score, evaluated by the calibration curve and the Hosmer-Lemeshow test. Finally, decision curve analysis (DCA) was performed to evaluate the net benefit of using the NEOS score to predict poor outcomes in children with anti-NMDAR encephalitis.

Statistical and ROC analyses were performed using SPSS version 23.0 (IBM, Armonk, NY, USA). Calibration curve and DCA were performed using rms and rmda packages, respectively, in R version 4.1.2 (http://www.r-project.org).

## Results

### Demographic Information and Clinical Characteristics

One hundred seventy-five children (101 females and 74 males) with anti-NMDAR encephalitis and a median age of 7.7 years (IQR: 4.7-10.7 years) were enrolled in this study. Seizures (38.9%), abnormal behaviors (36.4%), and movement disorders (12.3%) were the most common initial symptoms. **Table 2** presents their detailed clinical information. During the acute stage, all patients were treated with first-line immunotherapy. One hundred sixty-one patients (92.0%) were treated with a combination of intravenous methylprednisolone and immunoglobulin, of whom 4 patients (2.3%) received additional plasma exchange treatment. Four (2.3%) and 10 patients (5.7%) were exclusively treated with intravenous methylprednisolone or intravenous immunoglobulin, respectively. Sixteen patients (9.1%) received rituximab as second-line therapy. Only one patient was diagnosed with a teratoma and had tumor resection. At one year follow-up, 26 patients (14.9%) had a poor functional outcome (mRS > 2), while the remaining 149 patients (85.1%) had a good functional outcome (mRS ≤ 2). The PCPC scale was significantly correlated with patients’ mRS (Spearman r = 0.8345, 95% CI: 0.7814-0.8756, P < 0.001).

**Table 2:** Clinical characteristics of children with anti-NMDAR encephalitis.

Variables	All (n = 175)	Good functional status (n = 149)	Poor functional status (n =26)	P
Median age (years, IQR)	7.7 (4.7-10.7)	7.9 (5.4-10.9)	4.9 (1.9-9.8)	0.004
Sex (female, %)	101 (57.7)	83 (55.7)	18 (69.2)	0.198
Abnormal or psychiatric behavior (%)	151 (86.3)	135 (90.6)	16 (61.5)	<0.001
Speech dysfunction (%)	96 (54.9)	83 (55.7)	13 (50.0)	0.590
Seizures (%)	111 (63.4)	94 (63.1)	17 (65.4)	0.882
Movement disorder (%)	141 (80.6)	117 (78.5)	24 (92.3)	0.101
Decreased level of consciousness (%)	45 (25.7)	29 (19.5)	16 (61.5)	<0.001
Autonomic dysfunction (%)	22 (12.6)	19 (12.8)	3 (11.5)	>0.999
Sleep disorders (%)	98 (56.0)	89 (59.7)	9 (34.6)	0.017
Central hypoventilation (%)	10 (5.7)	5 (3.4)	5 (19.2)	0.007
ICU admission required (%)	11 (6.3)	6 (4.0)	5 (19.2)	0.012
Serum anti-NMDAR antibodies	141 (80.6)	123 (82.6)	18 (69.2)	0.113
Abnormal EEG (%)	157 (89.7)	131 (87.9)	26 (100)	0.079
Abnormal MRI (%)	82 (46.9)	62 (41.6)	20 (76.9)	0.001
CSF protein (mg/dL, IQR)	26 (17-36)	23 (15-33)	35 (24-57)	0.002
Increased CSF protein^*^ (%)	28 (16.0)	19(12.8)	9(34.6)	0.009
CSF WBC count > 20 cells/μL	57 (32.6)	49 (32.9)	8 (30.8)	0.832
No treatment within 4 weeks of symptom onset (%)	26 (14.9)	19 (12.8)	7 (26.9)	0.074
Treated with second-line immunotherapy (%)	16 (9.1)	14 (9.7)	2 (7.7)	>0.999
No clinical improvement after 4 weeks of treatment (%)	64 (36.6)	41 (27.5)	23 (88.5)	<0.001

*CSF protein levels > 45 mg/dL.

Among the five values of the NEOS score, patients with a poor outcome had more frequent ICU admissions (19.2% versus 4.0%, P = 0.012), abnormal MRI (76.9% versus 41.6%, P < 0.001) and no improvement 4 weeks after immunotherapy (88.5% versus 27.5%, P < 0.001). In contrast, there were no significant differences in CSF WBC or treatment interval between patients with different outcomes. Meanwhile, patients with bad outcomes were younger (4.9 versus 7.9 years, P = 0.004) and more frequently had decreased level of consciousness (61.5% versus 19.5%, P < 0.001), central hypoventilation (19.2% versus 3.4%, P = 0.007) and increased CSF protein (34.6% versus 12.8%, P = 0.009). In comparison, abnormal or psychiatric behavior (90.6% versus 61.5%, P < 0.001) and sleep disorder (59.7% versus 34.6%, P = 0.017) were more frequently observed in patients with good outcomes than in patients with bad outcomes (**Table 2**).

### Performance of the NEOS Score

**Figure 1:**
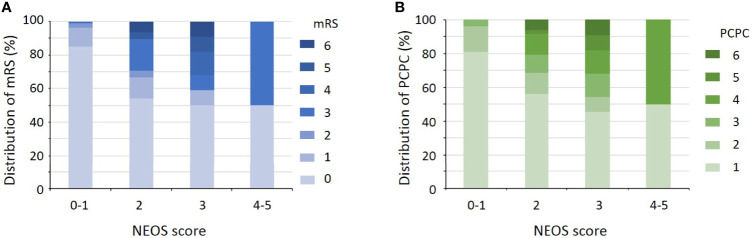
Distribution of the mRS **(A)** and PCPC scale **(B)** across the NEOS score.

The distribution of NEOS scores of the 175 patients were as follow: 41 patients with score = 0; 60 patients with score = 1; 48 patients with score = 2; 22 patients with score = 3; 4 patients with score = 4; and none of patients with score = 5. **Figure 1** shows the distribution of mRS and PCPC scale across NEOS score. The NEOS score was significantly associated with patients’ mRS (Spearman r = 0.3878, 95% CI: 0.2500-0.5103, P < 0.001) and PCPC scale (Spearman r = 0.3807, 95% CI: 0.2421-0.5041, P < 0.001). Moreover, patients with a higher NEOS score had a significantly higher risk (P < 0.001) of poor functional outcomes at one year follow-up (**Table 3**).

**Table 3:** Risks for poor functional outcome in patients with different NEOS scores.

NEOS score	Good functional status	Poor functional status	OR (95% confidence interval)	P
0-1	100 (99.0%)	1 (1.0%)	reference	–
2	34 (70.8%)	14 (29.2%)	41.176 (5.218-324.926)	< 0.001
3	13 (59.1%)	9 (40.9%)	69.231 (8.102-591.534)	< 0.001
4-5	2 (50.0%)	2 (50.0%)	100.000 (6.212-1609.854)	0.003

**Figure 2:**
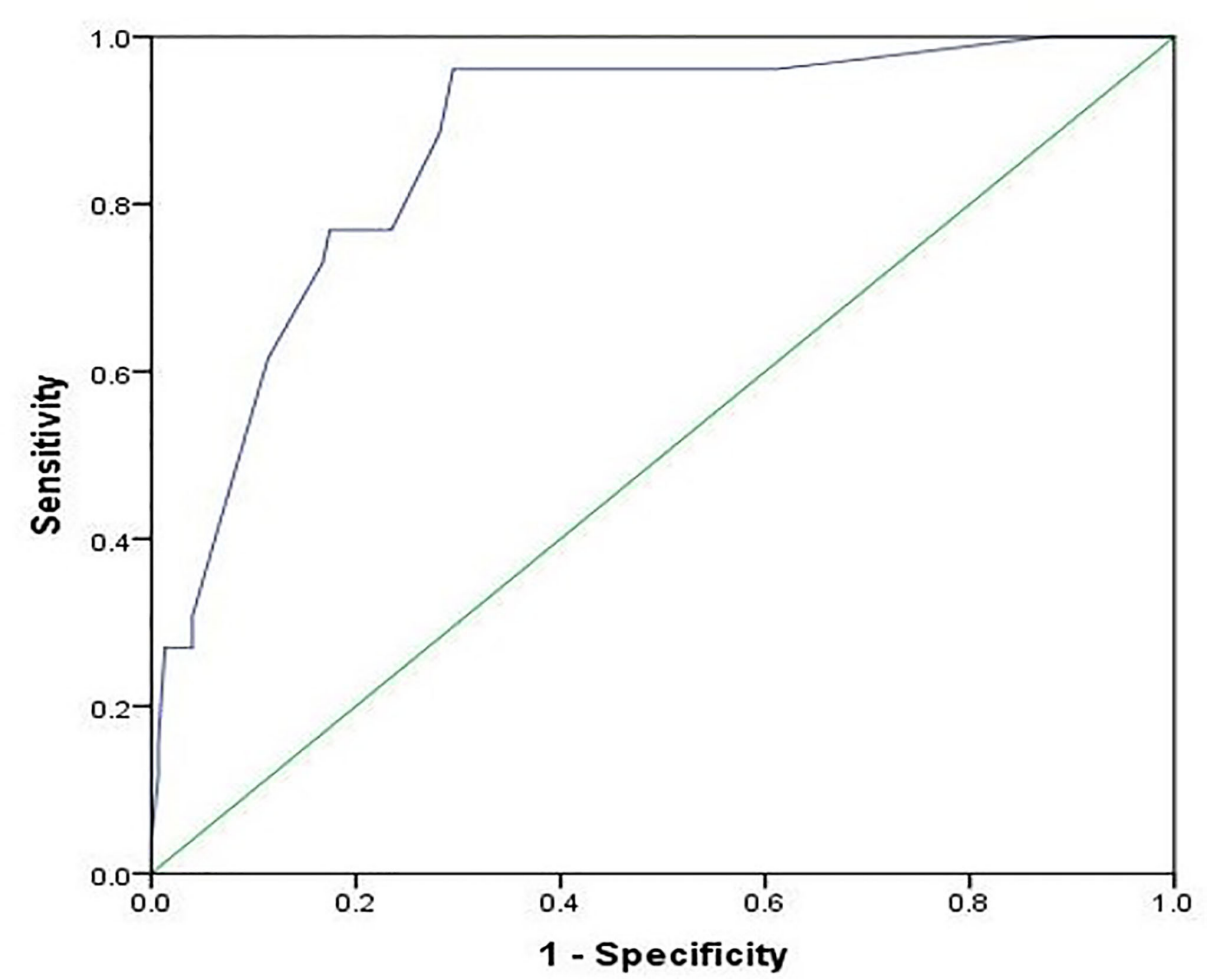
Receiver operating characteristic curve for the prediction of the one-year outcome using the NEOS score. The area under the curve is 0.870 (95% CI: 0.801-0.938, P < 0.001).

As presented in **Figure 2**, the NEOS score showed high discriminative power in predicting one-year functional status in this cohort of children (AUC = 0.870, 95% CI: 0.801-0.938, P < 0.001).

**Figure 3:**
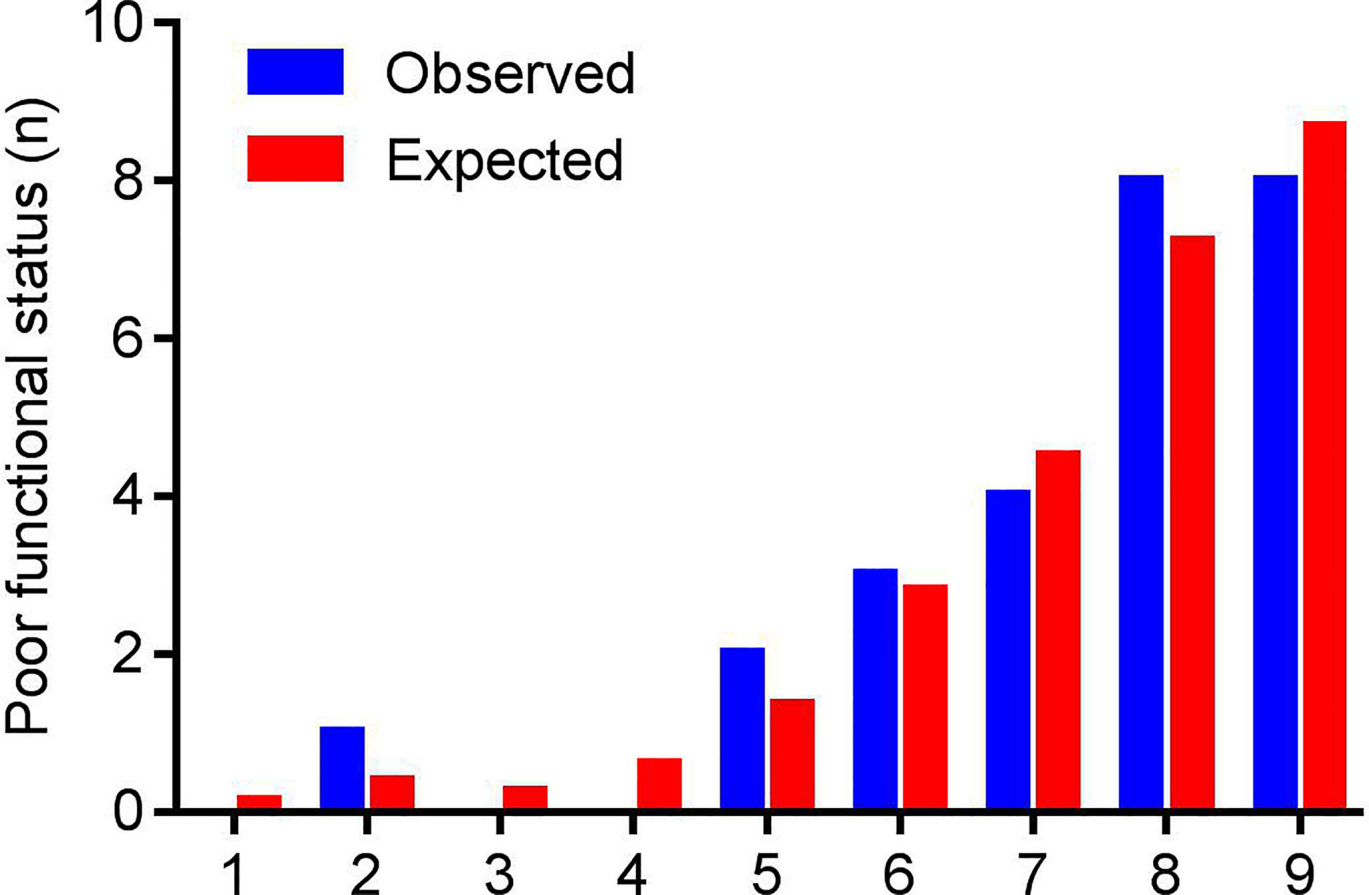
The results of the Hosmer-Lemeshow test. Patients were grouped based on the fitting probabilities (x axis). The blue column represents observed events (poor outcome at one year), and the red column represents predicted events of the NEOS score. There were no significant differences between observed and expected events (P = 0.912).

**Figure 4:**
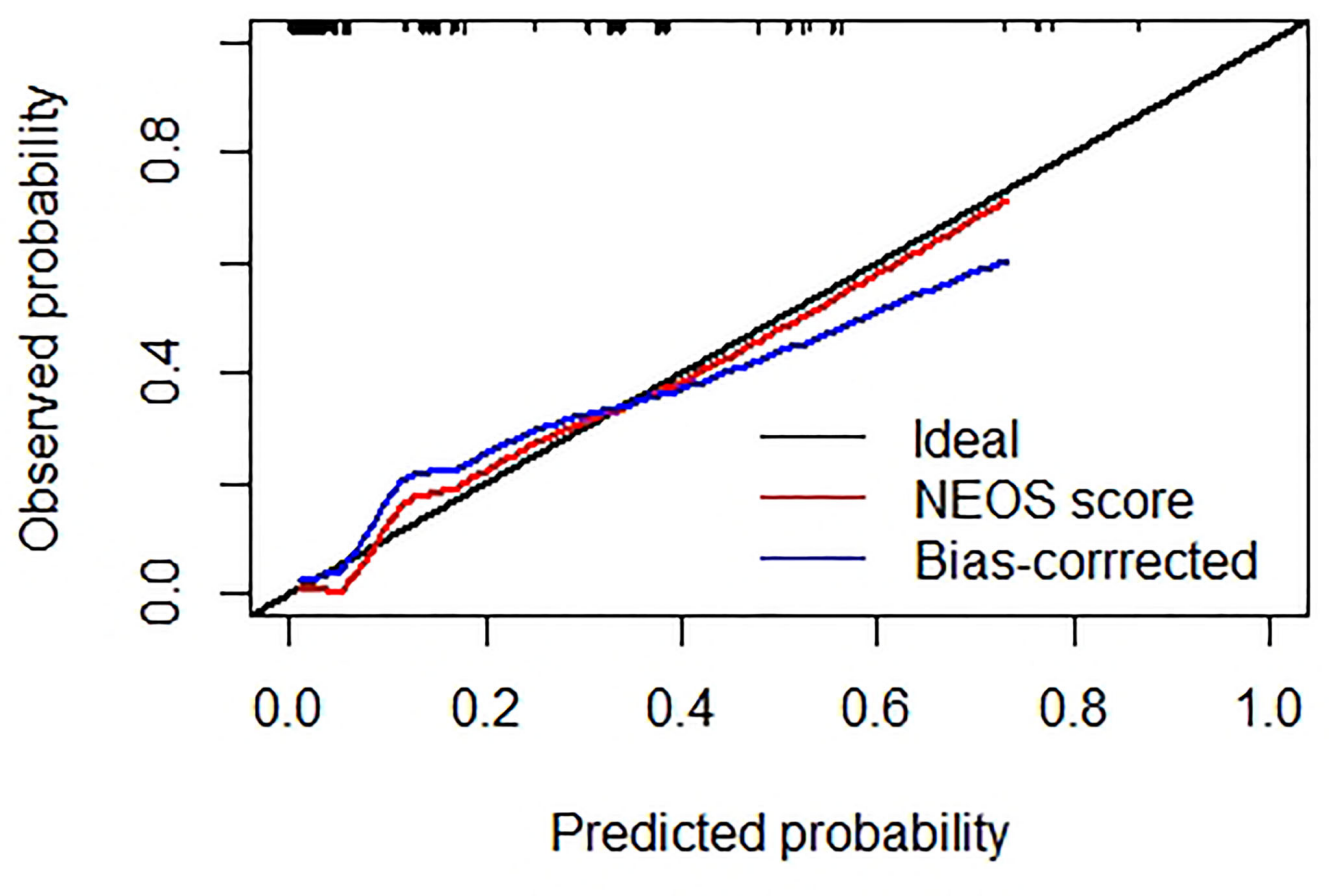
Calibration curve of the NEOS score. The black line represents the ideal situation, where the predicted probabilities are exactly equal to the observed probabilities. The red line represents the original calibration curve of the NEOS score. The blue line represents the bias-corrected curve by R software. Both curves show good consistency with the ideal situation (black line).

**Figure 3** shows the result of the Hosmer-Lemeshow test: the NEOS score showed a moderate consistency between the observed events and predicted events (P = 0.912). Meanwhile, the calibration curve of the NEOS score also fitted well with the ideal situation (**Figure 4**).

**Figure 5:**
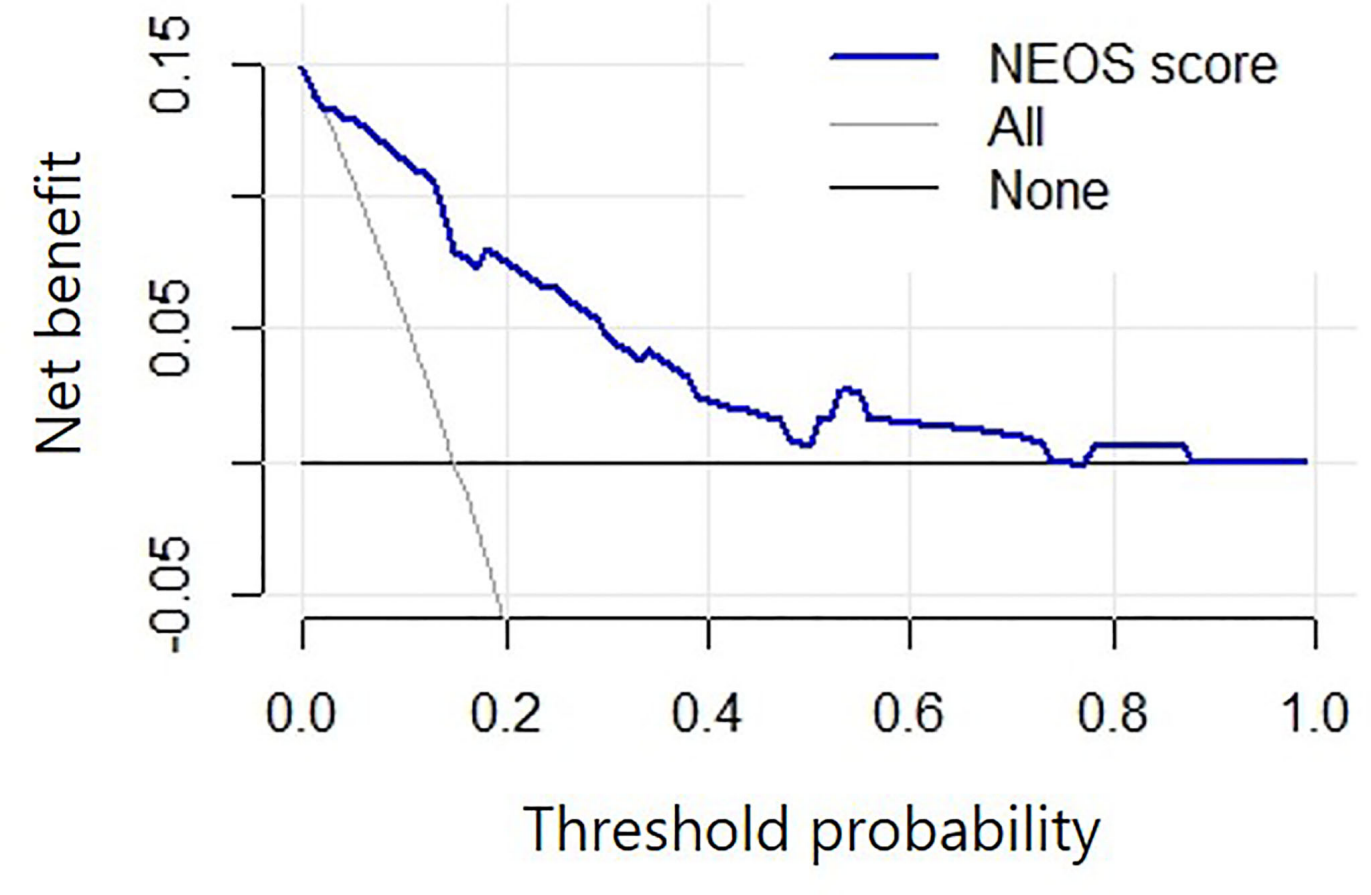
Decision curve analysis of the NEOS score. The horizontal black line (labeled “none”): no intervention for all patients, which indicates no net benefits. The slanted grey line (labeled “All”): all patients accept the intervention. The blue line shows that, compared to intervening with either no or all patients, a greater net benefit was achieved by using the NEOS score to predict the one-year outcome when the threshold probability was 1%-74%.

The DCA suggested that using the NEOS score to predict one-year outcome in this study may provide more net benefit than treating no patients or all patients when the threshold probability was 1%-74% (**Figure 5**). This result indicated that using the NEOS score to make clinical decisions about escalating treatment during clinical practice is beneficial.

## Discussion

In this study, we validated the performance of the NEOS score in children with anti-NMDAR encephalitis in a large cohort. Our results indicate that the NEOS score is valuable for predicting one-year functional outcomes with good clinical practicability in children with anti-NMDAR encephalitis.

The NEOS score was constructed by Balu et al. in 2019 from a cohort of 382 patients with anti-NMDAR encephalitis (5). In the original cohort, a higher NEOS score was significantly associated with the probability of poor functional status at one year. Its performance in Chinese patients was subsequently evaluated in a multicenter, prospective study by Peng et al. It showed good discrimination and calibration power with an AUC of 0.86 and a Pearson correlation coefficient of 0.53 (6). On the contrary, results from another study showed that the NEOS score was not associated with one-year outcomes of patients (13). However, this study only included a small sample size (20 patients) which may lead to substantial statistical bias. A modified NEOS score, using four of the five original values, was also associated with poor one-year outcomes (9). Although 57% of patients in the study by Peng et al. were juveniles (6), the performance of the NEOS score in pediatric patients has not yet been fully validated. A recent study evaluated the performance of the NEOS score in 30 children with anti-NMDAR encephalitis (14). However, the authors did not report the discrimination and calibration of the NEOS score, which are necessary for validating a predictive model. To our knowledge, this was the first study to comprehensively evaluate the performance of the NEOS score in pediatric cases.

Our results showed that the NEOS score was significantly associated with the mRS at one-year follow-up. A higher NEOS score indicated an increased risk for poor functional outcomes. This model could not only distinguish between patients with poor and good outcomes but also had good accuracy when compared to predicted probabilities from observed events. Further, children with anti-NMDAR encephalitis could benefit if the model was used to predict one-year outcomes within a wide range of threshold probability. In addition, to better evaluate the performance of NEOS scores in children, we also assessed patients’ outcomes using the PCPC scale. In line with the results of the mRS, a higher NEOS score was also associated with a larger PCPC scale at one-year follow-up. The first-line treatment regimen is already widely used (15, 16). However, the optimum timing to initiate second-line immunotherapy has not yet been determined and varies between different centers (9, 17). Overall, the timing of escalation to second-line immunotherapies should be individualized depending on the patient’s clinical status (18). Thus, our results supported that the NEOS score could improve the early identification of high-risk patients, who could then benefit from second-line or enhanced immunotherapy to improve their outcomes.

In this study, only 2% of patients had high NEOS scores of 4-5, much lower than in previous cohorts (5, 6). Meanwhile, when we refitted the NEOS score in our cohort, the CSF WBC and interval between onset and immunotherapy were not significantly related to increased risk for poor outcome. Moreover, a recent meta-analysis reported that abnormal findings on brain MRI were not associated with poor outcomes (9). This may be explained by the heterogeneity between different patients or treatment regimes but also may indicate that the clinical risk factors for poor outcomes differed between children and adults. Thus, these findings suggested that other reliable factors are required to modify this model further before we can generally apply it.

Besides the 5 items of the NEOS score, our results still presented several clinical features that were not consistent with the original study. First, the number of patients who had a poor outcome was much smaller than in the original cohort (5). The main reason could be that pediatric patients usually exhibit better outcomes than adults (19). Although the proportion of patients with poor outcomes was significantly lower than that of adults (7), it was in line with multiple previously-reported proportions in pediatric patients (20–24). Meanwhile, in line with a previous study on children (25), our study also indicated that younger patients were prone to have a poor outcome. Given the difficulties of detecting the behavioral change in little children (26), this might explain a significantly higher proportion of patients who had abnormal or psychiatric behavior.

In addition, we also found that increased CSF protein level was associated with poor outcomes. In the original NEOS study (5), Balu et al. reported that the CSF protein level was associated with poor functional status at one-year follow-up during univariate analysis but failed to be identified as an independent factor after multivariate regression analysis. Similar results were reported in a study conducted on children with anti-NMDAR encephalitis (25). Recently, more and more studies revealed that viral encephalitis could induce anti-NMDAR encephalitis (27). Children with anti-NMDAR encephalitis after viral encephalitis had significantly worse outcomes than patients with classical anti-NMDAR encephalitis (28). The precursor of viral encephalitis was an independent factor for poor outcomes in children with anti-NMDAR encephalitis (25). In our previous study, we found that patients with anti-NMDAR encephalitis after Japanese encephalitis had a significantly higher level of CSF protein when compared with classical anti-NMDAR encephalitis. However, there was no difference in CSF protein level between the acute stage of Japanese encephalitis and anti-NMDAR encephalitis (29). Thus, we speculated that increased CSF protein residual from the initial stage of viral encephalitis could be one of the reasons for this phenomenon. On the contrary, a recent study reported that children with good outcomes could have a relatively high level of CSF protein (23). Overall, risk factors for poor outcomes in children with anti-NMDAR encephalitis have substantial discrepancies among current studies. Other variables included sex, speech disorder, status epilepticus, central hypoventilation, decreased level of consciousness, resection of teratoma, and second-line therapy (20–23, 30–32). These results indicated dramatic heterogeneity among different centers. Thus, well-designed, multicenter research is imminently required to explore generally accepted predictors for the outcome of children with anti-NMDAR encephalitis.

This study had several limitations. Firstly, this study did not finely evaluate the cognitive outcome of children with anti-NMDAR encephalitis due to the restriction of the mRS and PCPC scale, which could limit the application of the NEOS score in children. Secondly, the number of children with poor outcomes in our study was too small to validate a predictive model comprehensively. Moreover, a single-center retrospective design exhibited unavoidable patient heterogeneity and selective bias. Finally, the original coefficients of this model in the developed cohort were not reported in the original research. Thus, statistical analysis was based on the refitted regression model in our cohort, which could influence the accuracy of our validation. Therefore, our results could not be generalized to all children with anti-NMDAR encephalitis across countries. The general application of the NEOS score in different regions still requires validation through further multicenter, large-scale studies.

In conclusion, the NEOS score is significantly associated with poor outcomes in children with anti-NMDAR encephalitis. It is a reliable tool to predict the one-year outcomes of children with anti-NMDAR encephalitis in clinical practice.

## Data Availability Statement

The raw data supporting the conclusions of this article will be made available by the authors, without undue reservation.

## Ethics Statement

The studies involving human participants were reviewed and approved by the ethics committees of the Children’s Hospital of Chongqing Medical University. Written informed consent to participate in this study was provided by the participants’ legal guardian/next of kin.

## Author Contributions

During the process of completing this research: LJ designed the study; HL, YL, YZ, LZ, JY, ZF, YJ, JW, ZY, and MC collected the patients’ clinical information. HL and YL performed the statistical analysis. HL drafted the first version of the manuscript. All authors participated in the critical review of the manuscript.

## Conflict of Interest

The authors declare that the research was conducted in the absence of any commercial or financial relationships that could be construed as a potential conflict of interest.

## Publisher’s Note

All claims expressed in this article are solely those of the authors and do not necessarily represent those of their affiliated organizations, or those of the publisher, the editors and the reviewers. Any product that may be evaluated in this article, or claim that may be made by its manufacturer, is not guaranteed or endorsed by the publisher.
